# Neurophysiological, metabolic and cellular compartments that drive neurovascular coupling and neuroimaging signals

**DOI:** 10.3389/fnene.2013.00003

**Published:** 2013-03-28

**Authors:** Andrea Moreno, Pierrick Jego, Feliberto de la Cruz, Santiago Canals

**Affiliations:** ^1^Instituto de Neurociencias, Consejo Superior de Investigaciones Científicas, Universidad Miguel HernándezSan Juan de Alicante, Spain; ^2^Centro de Estudios Avanzados de Cuba, Ministerio de Ciencia Tecnología y Medio AmbienteHabana, Cuba

**Keywords:** neurovascular coupling, fMRI, neurophysiology, astrocytes, glycolysis

## Abstract

Complete understanding of the mechanisms that coordinate work and energy supply of the brain, the so called neurovascular coupling, is fundamental to interpreting brain energetics and their influence on neuronal coding strategies, but also to interpreting signals obtained from brain imaging techniques such as functional magnetic resonance imaging. Interactions between neuronal activity and cerebral blood flow regulation are largely compartmentalized. First, there exists a functional compartmentalization in which glutamatergic peri-synaptic activity and its electrophysiological events occur in close proximity to vascular responses. Second, the metabolic processes that fuel peri-synaptic activity are partially segregated between glycolytic and oxidative compartments. Finally, there is cellular segregation between astrocytic and neuronal compartments, which has potentially important implications on neurovascular coupling. Experimental data is progressively showing a tight interaction between the products of energy consumption and neurotransmission-driven signaling molecules that regulate blood flow. Here, we review some of these issues in light of recent findings with special attention to the neuron-glia interplay on the generation of neuroimaging signals.

## INTRODUCTION

By the end of the nineteenth century, two scientists provided the first evidence in support of coordination between brain work and energy supply. Mosso (1881), on measuring brain pulsations over the right prefrontal cortex in a subject with abnormally thinned skull, reported increased pulsations when the subject performed a mathematical task. In 1890, Sherrington, using a more direct approach, showed that stimulation of the sensory nerves, or the medulla oblongata, produced an increase in brain blood pressure ( Roy and Sherrington, 1890). This hemodynamic response that accompanies brain activation was later found to also exist in pathological situations such as ischemia ( Ames et al., 1968). This vascular response was interpreted as a compensatory mechanism that fuels the brain either during increased energy expenditure or during restriction of metabolic substrate delivery.

Brain energy is mainly used for restoring the resting membrane potential of activated neurons ( Ames, 2000; Attwell and Laughlin, 2001). It is well established that the metabolic processes that fuel energy consumption are distributed in different cell types (neurons and glia) and subcellular compartments with predominantly either glycolytic or oxidative metabolisms. This segregation allows a functional compartmentalization of energy expenditure and is critical in understanding the mechanisms that coordinate brain work and energy supply, the so called neurovascular coupling. As such, is also fundamental in interpreting the signals obtained from brain imaging techniques as functional magnetic resonance imaging (fMRI).

Functional MRI based on blood oxygen level-dependent (BOLD) signal is the principal neuroimaging technique for basic and clinical research in humans. It is based on the paramagnetic nature of deoxygenated hemoglobin ( Pauling and Coryell, 1936) and the overcompensation of blood supply in response to brain activation that produces a net increase in oxygenated hemoglobin ( Fox and Raichle, 1986). This is accompanied by an enhancement of the MRI signal ( Ogawa and Lee, 1990; Ogawa et al., 1990).While the physical origin of the signal is clear, both the triggering mechanisms and its relation to neuronal activity are still controversial.

## NEUROPHYSIOLOGY

An important matter for neuroimaging is to understand which aspects of neuronal work are reflected in increased cerebral blood flow (CBF). Experiments simultaneously combining fMRI and electrophysiological recordings in the primary visual cortex of anesthetized monkeys showed that the imaging signal evoked by visual stimulation maximally correlates with the local field potential (LFP), an aggregate measure of synaptic and active dendritic currents ( Logothetis et al., 2001). Although the correlation of the BOLD signal was only slightly higher toward LFP compared with spiking activity (multiunit and single unit activity), the LFP signal was the only predictor of the hemodynamic response when long stimulation protocols that habituate spiking activity were used. Consistent with these findings were studies in the rat cerebellar cortex which convincingly showed that local CBF can indeed be dissociated from spiking activity while strongly correlated with LFPs ( Mathiesen et al., 1998, 2000; Thomsen et al., 2004).

**FIGURE 1:**
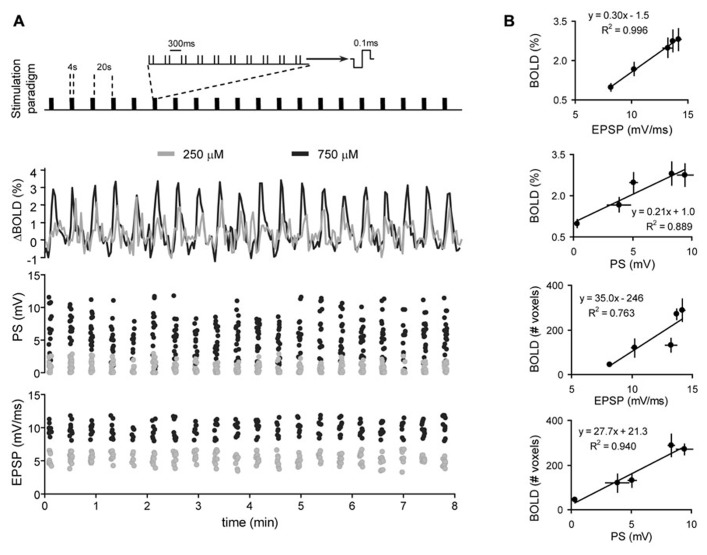
**Correlation of BOLD and electrophysiological signals.** **(A)** Simultaneous recordings of BOLD (middle panel) and electrophysiological signals (PS and EPSP; lower two panels, respectively) during a typical block-design protocol of electric-stimulation fMRI (upper panel). Two stimulation intensities in one animal are shown. **(B)** The amplitude of the BOLD signal in the dentate gyrus (%, two upper panels) or the volume of brain activated during perforant path stimulation (#, number of activated voxels, two lower panels) is plotted against the EPSP in the dentate gyrus or the corresponding PS. BOLD, EPSP and PS represent the mean ± SEM of all hemodynamic, synaptic and spiking responses, respectively, during a complete experiment (*n* = 5 experiments, collected in four different animals). Data is fitted with linear regression. Adapted with permission from Canals et al. (2009).

**FIGURE 2:**
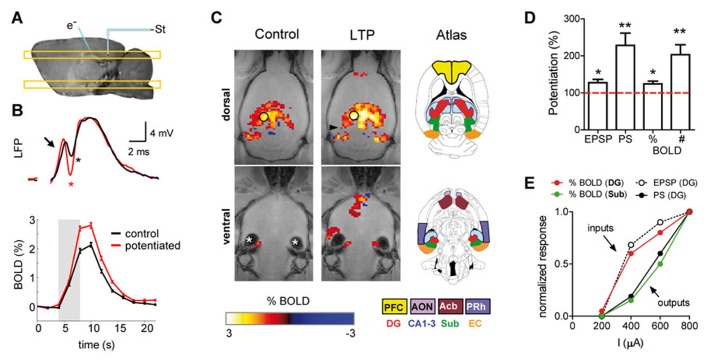
**Potentiation of the synaptic strength is accompanied by enhancement of the BOLD-fMRI signal.** **(A)** Schematic representation of the experimental preparation. The placement of the stimulating and recording electrodes is shown on a sagital T1-weighted image acquired with manganese-enhanced MRI. The location of the dorsal and ventral MRI slices shown in **(C)** is also illustrated. **(B)** LFP (upper panel) and BOLD signal time course (lower panel) recorded in the dentate gyrus during perforant pathway stimulation, before (black) and after (red) induction of long-term potentiation (LTP) of the synaptic strength. Arrow points toward the EPSP and asterisks mark the PS. **(C)** Functional maps thresholded and overlaid on horizontal FLASH anatomical scans, showing brain active areas during perforant path stimulation before (control) and after synaptic potentiation (LTP). New structures are recruited after LTP. Asterisks “*” mark image artifacts due to the ear channel. The color-coded scale represents positive and negative BOLD response in percent change from baseline. The position of the tip of the stimulation electrode is marked by the black arrowhead. The inset drawings, modified from the Paxinos and Watson rat brain atlas ( Paxinos and Watson, 2007), delineate different regions of interest. **(D)** Quantification of EPSP, PS and the amplitude of the BOLD signal (%) in the dentate gyrus, and the total number of activated fMRI voxels in the brain (#). **(E)** Electrophysiological and fMRI responses to different stimulation intensities measured in the dentate gyrus and the subiculum. Synaptic input (EPSP) and local BOLD signal amplitude in the dentate evolve together and diverge from the spiking output (PS) and its propagation to the subiculum. PFC, prefrontal cortex; AON, anterior olfactory nucleus; Acb, accumbens; PRh, perirhinal cortex; DG, dentate gyrus; Sub, subiculum; EC, entorhinal cortex. **(A–D)** adapted with permission from Canals et al. (2009).

Based on the above results, it has been argued that neuroimaging signals reflect the local processing of incoming neuronal activity to a particular area, rather than the output message being sent in outgoing efferent neuronal activity. Recent support of this view comes from combined fMRI-electrophysiology experiments, demonstrating that local synaptic plasticity modulates the amplitude of the BOLD signal ( Canals et al., 2009). In anesthetized rats, the dentate gyrus was activated with electrical microstimulation of the perforant pathway, while simultaneously recording electrophysiological and high resolution fMRI signals (**Figures 1** and **2**). Of note, in the hippocampus, the axial organization of the cellular elements, with a rather precise alignment of dendritic trees and somas, minimizes the cancellation of current sources form the LFP generators and facilitates the neurophysiological interpretation of the electrically-evoked field potentials, such as synaptic currents reflected in the excitatory post-synaptic potential (EPSP) and spiking activity in the population spike. Using this preparation, we showed that the glutamate-evoked post-synaptic currents were a precise predictor of BOLD signal amplitude, better than either the population spike or the electrical current used for stimulation ( Canals et al., 2008, 2009). This result has recently been confirmed in experiments combining electrophysiological recordings with hippocampal CBF measurements based on Laser-Doppler flowmetry ( Hamadate et al., 2011). Interestingly, the extension of the functional maps correlated better with the spiking activity than the EPSP recorded in the dentate gyrus ( Canals et al., 2009). This result indicated, perhaps not surprisingly, that while the local BOLD signal follows the synaptic input, the output (spiking) activity of a particular region predicts the activity propagation in the network connected to that region (**Figures 1** and **2E**).

It must be noted, however, that the local synaptic input of a volume of tissue relevant for the spatial resolution of fMRI (2–3 mm in human studies and 0.2–0.5 mm in small animal studies) is also contributed to by synaptic currents driven by recurrent spiking activity. Therefore, input and output activities are somehow mixed to some extent. What is the relative contribution to the BOLD signal of the synaptic inputs driven by recurrent spiking activity? In hippocampal preparations, when the strength of the synapses in the dentate gyrus was experimentally increased by means of a tetanic stimulation of the perforant pathway (long-term potentiation, LTP), the BOLD signal concomitantly increased ( Canals et al., 2009). Both, the EPSP and BOLD signal potentiations were of comparable magnitude and two to three times smaller than the spike potentiation. The dissociated evolution of the spiking activity vs. the BOLD signal and EPSP becomes even clearer in the small proportion of experiments in which, as originally described by Bliss and Lomo (1973), the tetanization of the perforant pathway is followed by an increase in the spiking probability without changing the EPSP (EPSP-to-spike or E-S potentiation). In these cases of increased tissue excitability, the local BOLD signal evoked by perforant path stimulation remains almost unaffected, like the EPSP, while the population spike amplitude increases two- to threefold (Benito et al., unpublished results). These results indicate that the effect of extrinsic synaptic inputs on the hyperemic response in the hippocampus outweigh the effect of synapses on recurrent axon collaterals, and suggest the intriguing possibility that not all glutamatergic synapses are equally suited to initiate a vascular response.

In summary, the above observations indicate that between the electrophysiological events constituting neuronal computations, glutamate evoked synaptic currents critically contribute to neuroimaging signals. It is intuitively appealing that the supply of energy substrates could be coupled to the process that, as mentioned earlier, consumes most of the energy used for neuronal signaling ( Ames, 2000; Attwell and Laughlin, 2001). However, the nature of the coupling mechanisms is under intense debate.

## METABOLISM vs. NEUROTRANSMISSION

Two major concepts have been put forward to mechanistically explain the coupling between neuronal activity and hyperemia. A classical view supports a feed-back mechanism in which the byproducts of energy expenditure act as signaling molecules to increase blood supply and restore energy buffers. In a more recent view, a feed-forward mechanism controlled by neurotransmitter-mediated signaling has a major role in CBF regulation. While neurotransmitter signaling is intrinsically correlated with energy consumption, the feed-forward model maintains that a causal link with CBF regulation only exists for the first. A large body of recent experimental data, mainly from *in vitro* preparations (see below), is shifting the field in favor of the feed-forward model ( Attwell et al., 2010; Petzold and Murthy, 2011). Both models, however, are mutually non-exclusive and may coexist in specific physiological states.

### METABOLIC FEED-BACK MECHANISM

Initial observations challenging the feed-back model came from studies showing that experimental manipulations of metabolic substrates such as O_2 _( Mintun et al., 2001; Lindauer et al., 2010) or glucose ( Powers et al., 1996) *in vivo* have little effect on CBF regulation. However, other metabolic byproducts such as adenosine can regulate CBF, linking the action of the Na^+^/K^+^ ATPase pump to local vasodilatation. Extracellular adenosine acting on A_2A_ receptors in vascular smooth muscle inhibits the arteriolar vasoconstriction mediated by the arachidonic acid (AA) metabolite 20-hydroxy-eicosatetraenoic acid (20-HETE; Gordon et al., 2008). This vasodilatory effect has been demonstrated in the cortex ( Ko et al., 1990) and the cerebellum ( Li and Iadecola, 1994) following neuronal stimulation. Adenosine effects on CBF regulation mediated by A_2B_ receptors have also been reported and involve interaction with Ca^2^^+^ signaling in astrocytes and probably AA metabolism ( Shi et al., 2008). A caveat in interpreting the source of extracellular adenosine is that ATP used as a gliotransmitter is also hydrolyzed to adenosine by extracellular ectonucleotidases ( Latini and Pedata, 2001). Therefore, depending on its origin, adenosine will couple CBF to energy consumption or neuronal signaling through glio-transmission. An additional unsolved question of adenosine-mediated functional hyperemia is the concomitant synaptic depressing effect that adenosine may exert on pre- and post-synaptic A_1_ receptors ( Greene and Haas, 1991), to which it binds with highest affinity ( Fredholm et al., 2001). The dual effect of adenosine may help maintain a safety energy level for neuronal integrity under conditions of transient energy limitations such as ischemia ( Dunwiddie and Masino, 2001; Canals et al., 2008), but it may interfere with neuronal computations in physiological conditions. The functional compartmentalization of adenosine release may constitute a solution to this potential problem ( Pelligrino et al., 2011).

One key energy metabolite in the brain which reinforces the feed-back model of neurovascular coupling is lactate. Important work on the special role of glycolysis in brain energy production provided the concept that glycolytic increase during brain activation ( Raichle and Mintun, 2006) results from the uptake of synaptically-released glutamate into astrocytes together with Na^+^ (Magistretti and Pellerin, 1999; Pellerin and Magistretti, 2004). In the astrocyte, glutamate is converted into glutamine and the excess Na^+^ is released to the extracellular space, both processes consuming ATP ( Erecinska and Silver, 1994). Refilling of the energy buffer seems to critically depend on glycolysis ( Raichle and Mintun, 2006), and glycolysis is linked to blood flow modulation through lactate. Accordingly, it has been convincingly shown that CBF response to an increase in neuronal activity is modulated by changes in the plasma lactate/pyruvate ratio in experimental animals ( Ido et al., 2001, 2004) and humans ( Mintun et al., 2004; Vlassenko et al., 2006). Accordingly, it has been recently shown that lactate indeed modulates the BOLD fMRI signal in the early visual cortex of non-human primates ( von Pfostl et al., 2012). These results and others ( Kasischke et al., 2004) support a direct metabolic (glycolytic) effect on CBF regulation and identify astrocytes as important players in the generation of neuroimaging signals.

Glutamate recycling is not the only mechanism linking astrocytes to functional hyperemia (see below), nor is metabolic compartmentalization of glycolytic enzymes restricted to astrocytes. Of potential functional (and imaging) relevance is also the discovery of glycolytic enzymes in the post-synaptic density of glutamatergic synapses ( Wu et al., 1997) and GABA receptors ( Laschet et al., 2004), potentially linking post-synaptic activations, glycolysis, and neuroimaging signals.

### NEUROTRANSMITTER-MEDIATED FEED-FORWARD SIGNALING

The feed-forward model is mainly represented by nitric oxide (NO) and AA metabolites released from neurons and glial cells as a consequence of glutamatergic neurotransmission. While vasoactive peptides and GABA (γ-aminobutyric acid) released by interneurons have been shown to contribute to the functional hyperemia in some systems ( Cauli et al., 2004; Kocharyan et al., 2008), and therefore contribute to the feed-forward neurovascular coupling mechanism, the experimental evidence accumulated to date is less abundant ( Lauritzen et al., 2012).

Synaptically-released glutamate acting on NMDA receptors increases post-synaptic Ca^2^^+^ levels and activates neuronal NO synthase (nNOS), which translates into NO release. NO mediates vasodilation in the brain, as repeatedly demonstrated in slice and *in vivo* preparations ( Busija et al., 2007). It has to be noted, however, that NO contribution to blood flow regulation presents regional differences in the brain ( Sokoloff et al., 1977). In the cortex (but not the cerebellum), while NO is required for functional hyperemia, it does not directly mediate the neuron-to-vessel signaling ( Lindauer et al., 1999). Its role in the cortex has been suggested to be the modulation of the AA metabolic pathways in astrocytes ( Attwell et al., 2010). There is strong evidence supporting blood flow regulation through the production and release of AA metabolites in response to synaptic glutamate. The supported mechanisms start with an mGluR-dependent increase in the astrocytic [Ca^2^^+^]i that activates phospholipase A_2_ and releases AA from membrane phospholipids. Subsequently, AA metabolites with vasodilatory activity, such as prostaglandins and epoxyeicosatrienoic acids, are produced and released. Interestingly, vasoconstrictions induced by increases in astrocytic [Ca^2^^+^]i have also been reported *in vitro* ( Mulligan and MacVicar, 2004; Metea and Newman, 2006) and are also mediated by the AA metabolite 20-HETE ( Behm et al., 2009). This conflicting result was elegantly explained by demonstrating that the metabolic state of the tissue ultimately determines the sign of the astrocytic control over vascular responses ( Gordon et al., 2008), with decreasing O_2_ concentrations favoring the production of vasodilatory responses. This interaction may therefore reflect the combination of feed-back and feed-forward mechanisms of blood flow regulation. Although conceptually attractive, it must be noted that the above *in vitro* results are challenged by previous ( Mintun et al., 2001) and recent ( Lindauer et al., 2010) studies showing no effect of O_2_ concentration on CBF regulation *in vivo* (see above).

A tight interaction between feed-back and feed-forward mechanism of neurovascular coupling starts to be clear as their respective counterparts are found to be closely related in the biochemical pathways supporting functional hyperemia. For example, the vasodilatory effect of adenosine at A_2A_ receptors is coupled to AA signaling pathway by interfering with 20-HETE vasoconstriction, a mechanism that also mediates part of the NO vasodilatory effect ( Roman, 2002). Furthermore, the mechanism linking lactate to vasodilation involves the inhibition of the prostaglandin transporter, increasing the extracellular concentration of PGE2 released from astrocytes and potentiating vasodilation ( Gordon et al., 2008). The vasodilatory effects of glutamate on astrocytes is reduced by blocking glutamate uptake ( Raichle, 1998; Bernardinelli et al., 2004), suggesting a metabolic contribution to the signaling effect. Therefore, activity-driven energy expenditure and neuronal or astrocytic-initiated signaling seem to be the two ends of the same rope. This may explain why inhibitory cocktails combining antagonists for the different pathways (AA, NO, adenosine) do not show additive blocking of functional hyperemia ( Koehler et al., 2006).

An additional level of interaction arises when considering that NO and AA have also been involved in the mechanism that strengthens synaptic currents after LTP induction and learning ( Schuman and Madison, 1991; Hardingham and Fox, 2006; DeCostanzo et al., 2010; Dachtler et al., 2011), protocols shown to increase the hyperemic response ( Canals et al., 2009; Hamadate et al., 2011). An intriguing possibility then is that the same set of molecules acting on converging signaling pathways is coordinating the strength of synaptic currents and energy consumption with the level of local blood supply. Whether a long-lasting increase in synaptic efficacy is accompanied by a similarly-lasting enhancement of neurovascular coupling efficacy is an important yet unsolved question, with potentially relevant implications on neuroimaging signal interpretation.

## ASTROCYTES

In light of the reviewed results and their strategic location between synapses and blood vessels, astrocytes have often been regarded as key players in the neurovascular coupling ( Ramón y Cajal, 1899). Astrocytes seem to be the compartment where many of the biochemical processes that determine the magnitude and direction of the vascular response to neuronal activation take place. Increases in astrocytic [Ca^2^^+^]i linked to AA metabolism is the principal mechanism thought to contribute to hyperemia. However, Ca^2^^+^ fluctuation in astrocytes not only occurs in response to neuronal activity (i.e., spontaneous events may also trigger astrocytic Ca^2^^+^ waves; Perea and Araque, 2005; Wang et al., 2009). Therefore, it has been suggested that a significant contribution to the neuroimaging signals may arise from the activation of astrocytes independently of neuronal signaling ( Wang et al., 2009). Nevertheless, a number of issues regarding Ca^2^^+^-mediated astrocyte-dependent neurovascular coupling still need to be clarified before a quantitative contribution of astrocytes to neuroimaging signals can be ascertained. First, functional hyperemia occurs less than 2 sec after the onset of the stimulation, whereas astrocytic Ca^2^^+^ elevation is slower, typically delayed by more than 2–3 sec ( Wang et al., 2006; Schummers et al., 2008). Based on this finding it was suggested that the astrocyte-Ca^2^^+^ response might be more important for sustaining the vasodilation during prolonged activation rather than as an initiating signal ( Koehler et al., 2009), an argument that has recently found some experimental support in simultaneous fMRI and fiber-optic Ca^2^^+^ recordings in rat neocortex ( Schulz et al., 2012). Second, and more importantly, neither spontaneous nor evoked [Ca^2^^+^]i increases in astrocytes are affected by ionotropic glutamate receptor antagonists such us CNQX (6-cyano-7-nitroquinoxaline-2,3-dione) and APV [(2R)-amino-5-phosphonovaleric acid; Takano et al., 2006; Thrane et al., 2012]. This pharmacological manipulation, however, eliminates both post-synaptic electrophysiological activity and the coupled vascular response.

## CONCLUDING REMARKS

Interactions between neuronal activity and CBF are largely compartmentalized. First, a functional compartmentalization that situates glutamatergic peri-synaptic activity and its electrophysiological events exists in close proximity to vascular coupling. Whether all glutamatergic synapses are equally suited for neurovascular coupling is an interesting yet unsolved question. In this direction, it is also important to acknowledge that heterogeneity in coupling mechanisms between different brain regions has already been reported and requires further attention. Second, the metabolic processes fueling peri-synaptic activity are partially segregated in glycolytic vs. oxidative compartments, with lactate production in response to increased neuronal activity as a key metabolite for energy supply, but also vascular coupling. A distinction between feed-back (metabolic) and feed-forward (signaling) mechanisms appears diffuse, since both mechanisms closely interact in the mediating biochemical pathways. An important issue to be clarified is whether a constant metabolic baseline and a constant neurovascular coupling efficiency can be assumed across brain states and synaptic plasticity. The quantitative value of neuroimaging signals may otherwise be affected by fluctuations in the reference baseline. Finally, a third level of segregation occurs at the cellular level, with astrocytic and neuronal compartments involved in vascular coupling through different but converging signaling pathways (NO, AA, adenosine, lactate/pyruvate ratio). Still, the actual relevance of astrocytes to neuroimaging signals needs to be clarified.

## Conflict of Interest Statement

The authors declare that the research was conducted in the absence of any commercial or financial relationships that could be construed as a potential conflict of interest.
